# Sequential Onset of Japanese Spotted Fever in a Parent-Child Household: A Familial Cluster of Two Cases

**DOI:** 10.7759/cureus.113842

**Published:** 2026-08-03

**Authors:** Natsumi Kamei, Tsuneaki Kenzaka, Ayako Kumabe, Naoya Mizutani, Tomohiro Hayashi

**Affiliations:** 1 Department of General Medicine, Public Toyooka Hospital, Toyooka, JPN; 2 Department of Community Medicine (Ibaraki), Graduate School of Medical and Dental Sciences, Institute of Science Tokyo, Tokyo, JPN; 3 Division of Community Medicine and Career Development, Kobe University Graduate School of Medicine, Kobe, JPN; 4 Department of Internal Medicine, Hyogo Prefectural Tamba Medical Center, Tamba, JPN

**Keywords:** case report, eschar, familial cluster, japanese spotted fever, rickettsia japonica, sequential onset, tick borne infection

## Abstract

Japanese spotted fever (JSF) is a tick-borne rickettsial infection caused by *Rickettsia japonica*. Although most cases occur sporadically, familial clusters may arise when multiple individuals are exposed to infected ticks in the same environment. In this report, we describe two sequential cases of JSF, representing, to our knowledge, the first reported parent-child familial cluster.

Case 1 involved a 61‑year‑old man who developed fever, rash, and multiple eschars one day after a tick bite. Laboratory testing revealed thrombocytopenia and elevated inflammatory markers. Intravenous minocycline, followed by oral therapy, resulted in clinical improvement. Polymerase chain reaction (PCR) testing of blood and eschar samples confirmed *R. japonica*.

Case 2 involved his 86‑year‑old mother, who developed fever and fatigue four days after the onset of her son’s symptoms. She had participated in the same outdoor activities and had continued to work in the field behind their home shortly before presentation. Physical examination revealed faint erythema, including the palms. Laboratory findings revealed mild hepatic dysfunction and elevated inflammatory marker levels. Oral minocycline was initiated, and PCR testing confirmed *R. japonica*. Both patients recovered without recurrence.

Given the feeding behavior of ticks, sequential bites from a single tick are rare. This case suggests exposure to multiple infected ticks, either inside the home or in the adjacent field. Clinicians should consider JSF in patients presenting with fever and rash when a cohabiting family member is diagnosed with JSF, even in the absence of clear outdoor exposure. Early antimicrobial therapy is essential to prevent severe complications.

## Introduction

Japanese spotted fever (JSF) is a rickettsial disease transmitted through the bites of ticks harboring *Rickettsia japonica *and characterized by fever, rash, and eschar [1]. JSF was first described by Mahara et al. [2] in 1984; since then, more than 3,000 cases have been reported by the National Institutes of Health in Japan [3]. JSF is endemic predominantly in western Japan, and the number of reported cases has been increasing in recent years [4]. Clinically, JSF typically presents with the triad of fever, rash, and eschar. Liver function abnormalities are also commonly observed [4]. However, because the three symptoms do not always occur simultaneously, early diagnosis can be challenging [4]. Delayed diagnosis and treatment may result in severe complications, including disseminated intravascular coagulation, multiple organ failure, and death [4]. Therefore, prompt recognition of JSF and early administration of tetracycline antibiotics are essential to improve patient outcomes.

In Japan, ticks that transmit *R. japonica *include *Haemaphysalis flava*, *Dermacentor taiwanensis*, and *Ixodes ovatus* [5]. When wild animals such as deer, boar, and small mammals are infected, ticks acquire rickettsiae through blood feeding [6]. The rickettsiae multiply within the tick and persist transstadially across developmental stages, namely, the egg, larva, nymph, and adult [6]. When an infected tick attaches to human skin and begins blood feeding, rickettsiae invade the human body via the salivary glands, resulting in infection [6]. Ticks primarily inhabit grasslands, including mountainous areas and river basins, where they feed on the blood of mammals and birds. In regions with high tick populations, tick bites commonly occur during agricultural work and outdoor activities [7].

Although JSF typically occurs sporadically, familial clusters have occasionally been reported, presumably because family members are exposed to the same tick-infested environment [3]; however, such clusters are rare. Thus, we aimed to document a familial cluster of JSF, involving a parent and child who developed the disease almost simultaneously.

## Case presentation

Case 1

A 61-year-old Japanese man presented with fever and rash. His medical history included diabetes mellitus, hypertension, and dyslipidemia. In early August 2023, the patient noticed bite marks on the right anterior and left posterior lower legs. On day 1, he was diagnosed with tick bites at a local clinic. On day 2, he developed a fever of 38°C and general fatigue, followed by the appearance of a rash. On day 6, he visited the hospital emergency department and was admitted.

Regarding outdoor activities, he had been cutting and burning weeds in a field behind his house, along with his mother (Case 2). He reported no other history of working in mountainous or grassy areas. Although he had previously worked on rice-field ridges, he reported wearing long pants and boots while working.

**Figure 1:**
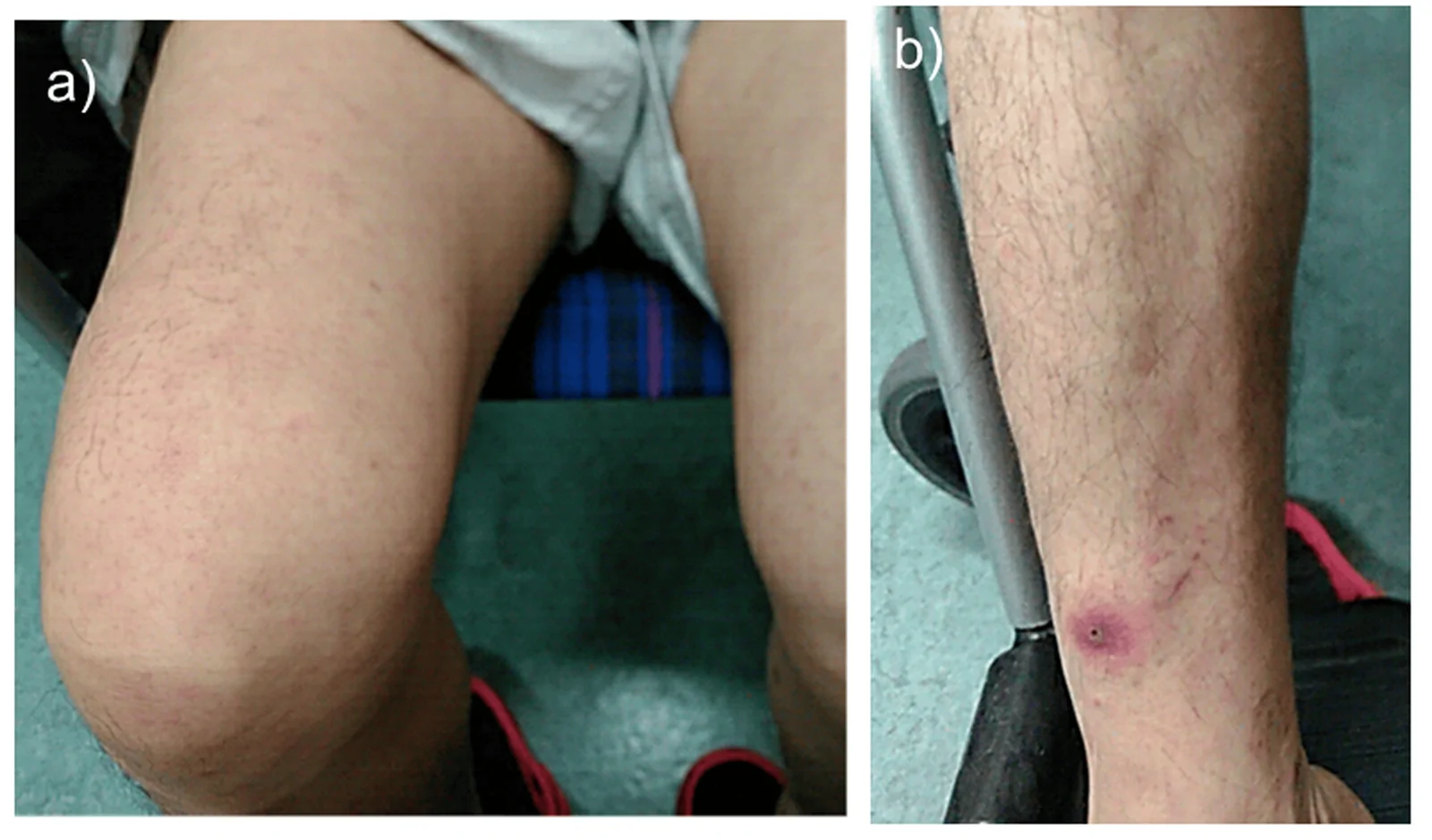
In Case 1, the right thigh (a) and right lower leg (b) are shown. Erythema is present, and eschars are identified on the right lower leg.

On presentation, the patient was alert and oriented, with blood pressure 89/52 mmHg, heart rate 101 beats/min, respiratory rate 18 breaths/min, body temperature 37.9°C, and oxygen saturation 98% on room air. Superficial lymphadenopathy was not observed, and cardiac and respiratory examination results were unremarkable. No joint tenderness, redness, or warmth was observed. Several eschars were observed on both lower legs. Erythematous rashes were present on the neck, trunk, and extremities but not on the palms (Figure 1).

The laboratory findings on admission are shown in Table 1.

**Table 1:** Laboratory data upon admission of Case 1.

Parameter	Recorded value	Standard value
White blood cell count	6,000/µL	4,500-7,500/µL
Neutrophils	84.4%	42-74%
Lymphocytes	10.5%	18-50%
Hemoglobin	16.6 g/dL	11.3-15.2 g/dL
Platelet count	10.6 × 10^4^/µL	13-35 × 10^4^/µL
Prothrombin time / International normalized ratio	1	0.80-1.20
Activated partial thromboplastin time	31.7 s	26.9-38.1 s
C-reactive protein	10.3 mg/dL	≤0.60 mg/dL
Total protein	6.9 g/dL	6.9-8.4 g/dL
Albumin	3.7 g/dL	3.9-5.1 g/dL
Total bilirubin	0.8 mg/dL	0.2-1.2 mg/dL
Aspartate aminotransferase	40 U/L	11-30 U/L
Alanine aminotransferase	37 U/L	4-30 U/L
Lactase dehydrogenase	254 U/L	109-216 U/L
Creatine kinase	50 U/L	40-150 U/L
Blood urea nitrogen	17.3 mg/dL	8-20 mg/dL
Creatinine	0.76 mg/dL	0.63-1.03 mg/dL
Sodium	133 mEq/L	136-148 mEq/L
Potassium	3.5 mEq/L	3.6-5.0 mEq/L
Chloride	95 mEq/L	98-108 mEq/L
Glucose	247 mg/dL	70-109 mg/dL
Hemoglobin A1c	9.1%	5.6-5.9%

The platelet count decreased (106,000/μL), although coagulation parameters were within normal limits. Aspartate aminotransferase (AST) and alanine aminotransferase (ALT) levels were mildly elevated. Inflammatory markers were elevated, with increased C-reactive protein (CRP) levels. Based on the presence of fever and rash, JSF or scrub typhus was suspected, and the patient was admitted. Minocycline 100 mg was administered intravenously twice daily. His blood pressure improved promptly after fluid resuscitation and antibiotic therapy.

On day 10, treatment was switched to oral minocycline (100 mg twice daily), and antibiotics were continued for eight days. The patient gradually became afebrile, with defervescence by day 11 and disappearance of the rash by day 12. Polymerase chain reaction (PCR) testing of blood and skin crusts obtained at admission detected *R. japonica*, confirming the diagnosis of JSF. The patient was discharged on day 13 of illness and has since remained recurrence-free for more than two years.

Case 2

An 86-year-old Japanese woman presented with a fever. The patient was the mother of the patient in Case 1, lived with him, and was independent in activities of daily living. She had a history of surgically treated left breast cancer. Her comorbidities included diabetes mellitus and dyslipidemia. On day 9, three days after Case 1 was hospitalized, she developed general fatigue. On day 12, she developed a fever of 39.2°C and a rash. The following day (day 13), she presented to the hospital emergency department and was admitted. Regarding outdoor exposure, she had mowed and burned grass with her son (Case 1) in a field behind their house. She had also worked in the same field two days before presentation, five days after the patient in Case 1 had been hospitalized. She reported no other recent exposure to mountainous or grassy areas.　

**Figure 2:**
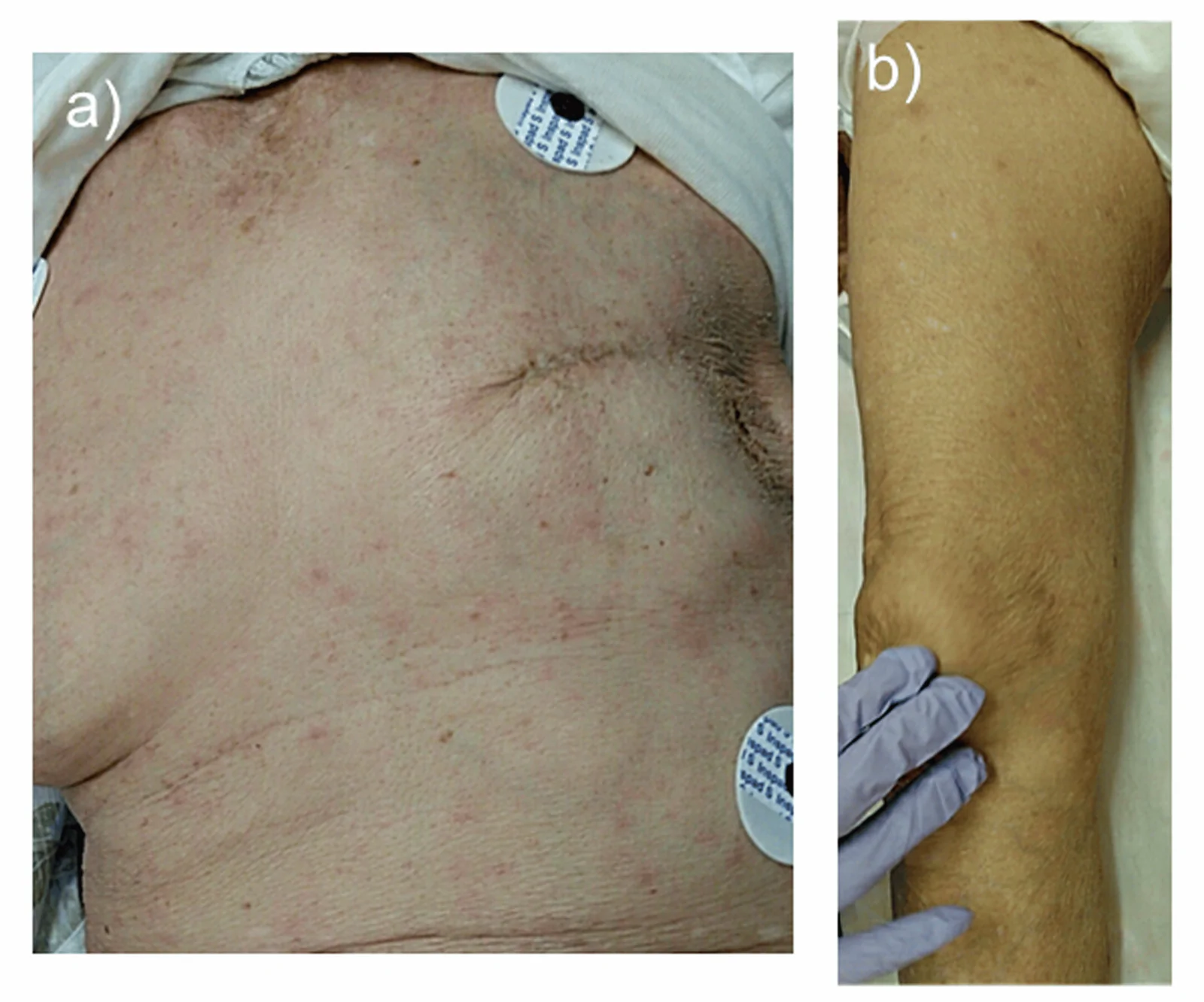
In Case 2, the trunk (a) and right lower limb (b) are shown. A faint erythema is noted.

At presentation, the patient was alert and oriented, with blood pressure 93/46 mmHg, heart rate 80 beats/min, respiratory rate 18 breaths/min, body temperature 37.0°C (after antipyretic use), and oxygen saturation 96% on room air. Superficial lymphadenopathy was not observed, and cardiac and respiratory examination results were unremarkable. No joint tenderness, erythema, or warmth was observed. Faint erythematous rashes were observed on the trunk and extremities, including the palms (Figure 2).

The laboratory findings on admission are shown in Table 2.

**Table 2:** Laboratory data upon admission of Case 2.

Parameter	Recorded value	Standard value
White blood cell count	8,200/µL	4,500-7,500/µL
Neutrophils	88.1%	42-74%
Lymphocytes	7.1%	18-50%
Hemoglobin	12.5 g/dL	11.3-15.2 g/dL
Platelet count	14.5 × 10^4^/µL	13-35 × 10^4^/µL
Prothrombin time / International normalized ratio	1.09	0.80-1.20
Activated partial thromboplastin time	28.6 s	26.9-38.1 s
C-reactive protein	7.12 mg/dL	≤0.60 mg/dL
Total protein	5.6 g/dL	6.9-8.4 g/dL
Albumin	3.1 g/dL	3.9-5.1 g/dL
Total bilirubin	0.8 mg/dL	0.2-1.2 mg/dL
Aspartate aminotransferase	75 U/L	11-30 U/L
Alanine aminotransferase	34 U/L	4-30 U/L
Lactase dehydrogenase	488 U/L	109-216 U/L
Creatine kinase	443 U/L	40-150 U/L
Blood urea nitrogen	17 mg/dL	8-20 mg/dL
Creatinine	0.79 mg/dL	0.63-1.03 mg/dL
Sodium	131 mEq/L	136-148 mEq/L
Potassium	4.3 mEq/L	3.6-5.0 mEq/L
Chloride	98 mEq/L	98-108 mEq/L
Glucose	195 mg/dL	70-109 mg/dL
Hemoglobin A1c	6.9%	5.6-5.9%

Platelet count and coagulation parameters were within the normal range. AST levels were mildly elevated, and an increase in CRP levels was observed. Based on the clinical course of Case 1, JSF was suspected.

Antimicrobial therapy with oral minocycline (100 mg, twice daily) was initiated. Her blood pressure promptly improved after initiating fluid resuscitation and antimicrobial therapy. She became afebrile on day 16, and the erythema on the lower legs resolved. PCR testing of blood and eschar samples obtained upon admission revealed *R. japonica*, confirming the diagnosis of JSF. Minocycline was administered for eight days, and the patient was discharged on day 20. The patient has remained recurrence-free for more than two years.

The timelines for the two cases are presented in Table 3.

**Table 3:** Timeline of exposure and clinical course of the two family members with Japanese spotted fever. Both patients participated in weed-cutting and burning activities before symptom onset. The son developed Japanese spotted fever first, whereas the mother developed symptoms seven days later after continuing to work in the same field. PCR confirmed *Rickettsia japonica* infection in both patients. PCR: polymerase chain reaction

Date (August 2023)	Case 1 (Son, 61 y)	Case 2 (Mother, 86 y)
Early Aug	Weed-cutting and burning with his mother in the field behind their house	Weed-cutting and burning with her son in the same field
Day 1	He noticed an eschar and visited a local clinic, where he was diagnosed with tick bites	-
Day 2	Fever (38°C), fatigue, rash	-
Day 6	Emergency department → hospitalization; Intravenous minocycline started	-
Day 9	-	General fatigue began
Day 10	Switched to oral minocycline	-
Day 12	-	Fever (39°C), rash
Day 13	Polymerase chain reaction positive for *Rickettsia japonica* Discharge	Emergency department → Hospitalization; Oral minocycline started
Day 18	-	Polymerase chain reaction positive for *Rickettsia japonica*
Day 20	-	Discharge

## Discussion

In this report, we present an extremely rare familial occurrence of JSF. Because JSF is not transmitted from person to person, familial clustering is uncommon. All previously reported cases of familial JSF have involved multiple family members who developed the disease owing to shared environmental exposure. To the best of our knowledge, based on a PubMed search of previously reported familial clusters of JSF, all documented cases have involved married couples. Therefore, this appears to be the first reported case of familial JSF occurring between family members other than a married couple [3,8,9].

JSF is characterized by fever, rash, and eschar [1]. The incubation period ranges from 2 to 10 days [1]; in some cases, an eschar cannot be identified [3]. Although the prognosis is generally favorable, delayed treatment may lead to severe complications, such as hemophagocytic syndrome and multiple organ failure, which can be fatal [10,11]. Delay in treatment has been identified as a poor prognostic factor. Furthermore, the absence of an identifiable eschar may contribute to the delayed diagnosis of rickettsial diseases, and this is also associated with poor outcomes [12-14]. Therefore, prompt initiation of antimicrobial therapy is strongly recommended when JSF is suspected based on clinical features, such as rash, thrombocytopenia, and liver dysfunction.

Ticks feed on blood for several days to approximately one week and can only feed two or three times during their lifetime [15]. Although human-to-human transmission of JSF does not occur, several familial cases have been reported. Table 4 summarizes previously reported familial cases of JSF that were identified [3,8,9].

**Table 4:** List of past intrafamilial transmissions of Japanese spotted fever. yo: years old

List of family infections	Reference	Family members and their ages	Interval between onset times	Precipitating factors
1	[3]	Married couple (husband 65 yo, wife 59 yo)	Concurrent onset	Camp
2	[4]	Married couple (husband, age unknown, wife 76 yo)	Concurrent onset	No outdoor activity experience
3	[5]	Married couple (husband 80 yo, wife 74 yo)	Onset four days later	Home vegetable garden, living near grassland
Our case	-	Mother and son (mother 86 yo, son 61 yo)	Onset seven days later	Inside the house or the field behind it

In all cases, the affected individuals were married couples who developed symptoms either simultaneously or within a few days of each other. By contrast, the present cases were a parent and child, representing the first reported familial occurrence of JSF outside of a spousal relationship. Moreover, the interval between disease onset in the two family members was seven days, which was longer than that described previously.

Some cases of JSF have been reported in individuals without a clear history of outdoor activity [5]. In mountainous residential areas, ticks may inhabit household environments and cause bites indoors [15]. Therefore, JSF can occur even in the absence of apparent outdoor exposure in regions where ticks carrying *R. japonica* are endemic.

In the present cases, considering the biological characteristics of ticks, it is unlikely that a single tick bit both patients sequentially. Although the exact source of exposure remains uncertain, a plausible hypothesis is that multiple ticks harboring *R. japonica* were present and caused successive bites, either inside the house or in the adjacent field, resulting in this familial cluster.

Therefore, when a cohabiting family member or an individual who shares outdoor activities is diagnosed with JSF, clinicians should maintain a high index of suspicion for JSF in other family members presenting with fever and rash.

## Conclusions

We encountered two sequential cases of JSF within the same family, underscoring the risk of familial or shared environmental exposure. In regions where ticks harboring *R. japonica *are endemic, obtaining detailed medical and exposure histories, including possible familial occurrence, is essential. Prompt initiation of antimicrobial therapy when JSF is suspected, particularly in patients presenting with fever and rash, is crucial for preventing severe complications.
